# ARID1A serves as a receivable biomarker for the resistance to EGFR-TKIs in non-small cell lung cancer

**DOI:** 10.1186/s10020-021-00400-5

**Published:** 2021-10-29

**Authors:** Dantong Sun, Fei Teng, Puyuan Xing, Junling Li

**Affiliations:** National Cancer Center/National Clinical Research Center for Cancer/Cancer Hospital, Chinese Academy of Medical Sciences and Peking Union Medical College, Beijing, 100021 China

**Keywords:** Switch/sucrose nonfermenting, ARID1A, EGFR-TKIs, Resistance, NSCLC

## Abstract

ARID1A is a key component of the SWI/SNF chromatin remodeling complexes which is important for the maintaining of biological processes of cells. Recent studies had uncovered the potential role of *ARID1A* alterations or expression loss in the therapeutic sensitivity of cancers, but the studies in this field requires to be further summarized and discussed. Therefore, we proposed a series of mechanisms related to the resistance to EGFR-TKIs induced by *ARID1A* alterations or expression loss and the potential therapeutic strategies to overcome the resistance based on published studies. It suggested that *ARID1A* alterations or expression loss might be the regulators in PI3K/Akt, JAK/STAT and NF-κB signaling pathways which are strongly associated with the resistance to EGFR-TKIs in NSCLC patients harboring sensitive *EGFR* mutations. Besides, *ARID1A* alterations or expression loss could lead to the resistance to EGFR-TKIs via a variety of processes during the tumorigenesis and development of cancers, including epithelial to mesenchymal transition, angiogenesis and the inhibition of apoptosis. Based on the potential mechanisms related to ARID1A, we summarized that the small molecular inhibitors targeting ARID1A or PI3K/Akt pathway, the anti-angiogenic therapy and immune checkpoint inhibitors could be used for the supplementary treatment for EGFR-TKIs among NSCLC patients harboring the concomitant alterations of sensitive *EGFR* mutations and *ARID1A*.

## Introduction

Lung cancer ranks first among all malignancies in cancer-related mortality, and the 5-year overall survival (OS) is lower than 20% in China, which causes a serious situation for public health (Allemani et al. 2018). Besides the small cell lung cancer (SCLC), non-small-cell lung cancer (NSCLC) consists of approximately 85% of all lung cancer cases (Hou et al. 2019a) and the novel therapeutics had achieved a better response than before. NSCLC patients are easily detected harboring cancer genome with highly instability, especially for Asians. Targeted therapeutics based on the driver mutations of NSCLC, such as mutations of *epidermal growth factor receptor* (*EGFR*) (Santoni-Rugiu et al. 2019) and rearrangement of anaplastic lymphoma kinase (ALK) (Golding et al. 2018), have significantly prolonged the survival of NSCLC patients. Unfortunately, NSCLC patients harboring sensitive *EGFR* mutations still could develop the resistance to EGFR-tyrosine kinase inhibitors (TKIs) primarily or secondarily, which leads to treatment failure. According to previous studies, varieties of mechanisms have been proven to be associated with the resistance to EGFR-TKIs, such as the pre-existing T790M mutation of *EGFR* (Inukai et al. 2006; Lee et al. 2014) which causes the primary resistance to first generation of EGFR-TKIs, *insulin-like growth factor 1 receptor* (*IGF1R*) mutation (Sharma et al. 2010), *MET* amplification (Turke et al. 2010), *hepatocyte growth factor* (*HGF*) mutation (Yano et al. 2008) and mutations leading to sustained activated signaling in other pathways, including the PI3K/AKT pathway (Tan et al. 2015), which causes the resistance to both first generation and third generation of EGFR-TKIs. Nevertheless, still a proportion of NSCLC patients harboring sensitive *EGFR* mutations might develop the resistance to EGFR-TKIs via unknown mechanisms. Hence, it is of great significance to explore the potential mechanism related to the resistance to EGFR-TKIs.

Switch/sucrose nonfermenting (SWI/SNF) chromatin remodeling complexes perform essential roles in a series of biological processes, including DNA replication, gene expression and cell differentiation (Wang et al. 2004; Zhang et al. 2014). In addition, molecules of SWI/SNF chromatin remodeling complexes have been found to be dysregulated frequently in various cancer types (Huang et al. 2015). A variety of subunits of the SWI/SNF chromatin remodeling complexes had been identified, including AT-rich interactive domain 1A (ARID1A) (Michel et al. 2018; Mashtalir et al. 2018) and so on. ARID1A is a key component of the SWI/SNF chromatin remodeling complexes (ARID1A is the key subunit of BAF, while BAF is the main assembly of SWI/SNF complexes) that can bind DNA in a non-sequence-specific manner via alternating the tensity of nucleosome and are involved in the processes of DNA repair and stabilization (Wang et al. 2004; Reisman et al. 2009) which are closely related to the cell fate decision (Pagliaroli and Trizzino 2021) and also serve as a multifunctional regulator of subplate-dependent guidance mechanisms essential to cortical circuit wiring (Doyle et al. 2021). Alterations in *ARID1A* may be diverse and have been observed in a variety of cancer types, including urothelial carcinoma (Dugas et al. 2019), gastric cancer (Kim et al. 2019) and lung cancer (Huang et al. 2015; Naito et al. 2019) and the variants of *ARID1A* gene could also be detected through liquid biopsy even for cancers of unknown primary (Laprovitera et al. 2021) as well. Previous studies had demonstrated the essential role of ARID1A in carcinogenesis and cancer development. The loss of ARID1A, which usually lead to the resultant loss of intact BAF, would causes the rapid carcinogenesis across tissues (Wang et al. 2020a). Meantime, loss of ARID1A was found to be associated with the poor prognosis of a variety of cancers including hepatocellular carcinoma (HCC) (Yim et al. 2020) and endometrial carcinoma (EC) (Leo et al. 2021). Researchers had also focused on the role of ARID1A in cancer therapeutics. Andrade confirmed that the intact ARID1A is important in maintaining the sensitivity to radiotherapy in breast cancer via suppressing the accumulation of DNA double-strand breaks (DSBs) caused by radiation (Andrade et al. 2019). However, whether ARID1A plays a role in the resistance to EGFR-TKIs remains unclear and requires to be further elucidated.

**Fig. 1:**
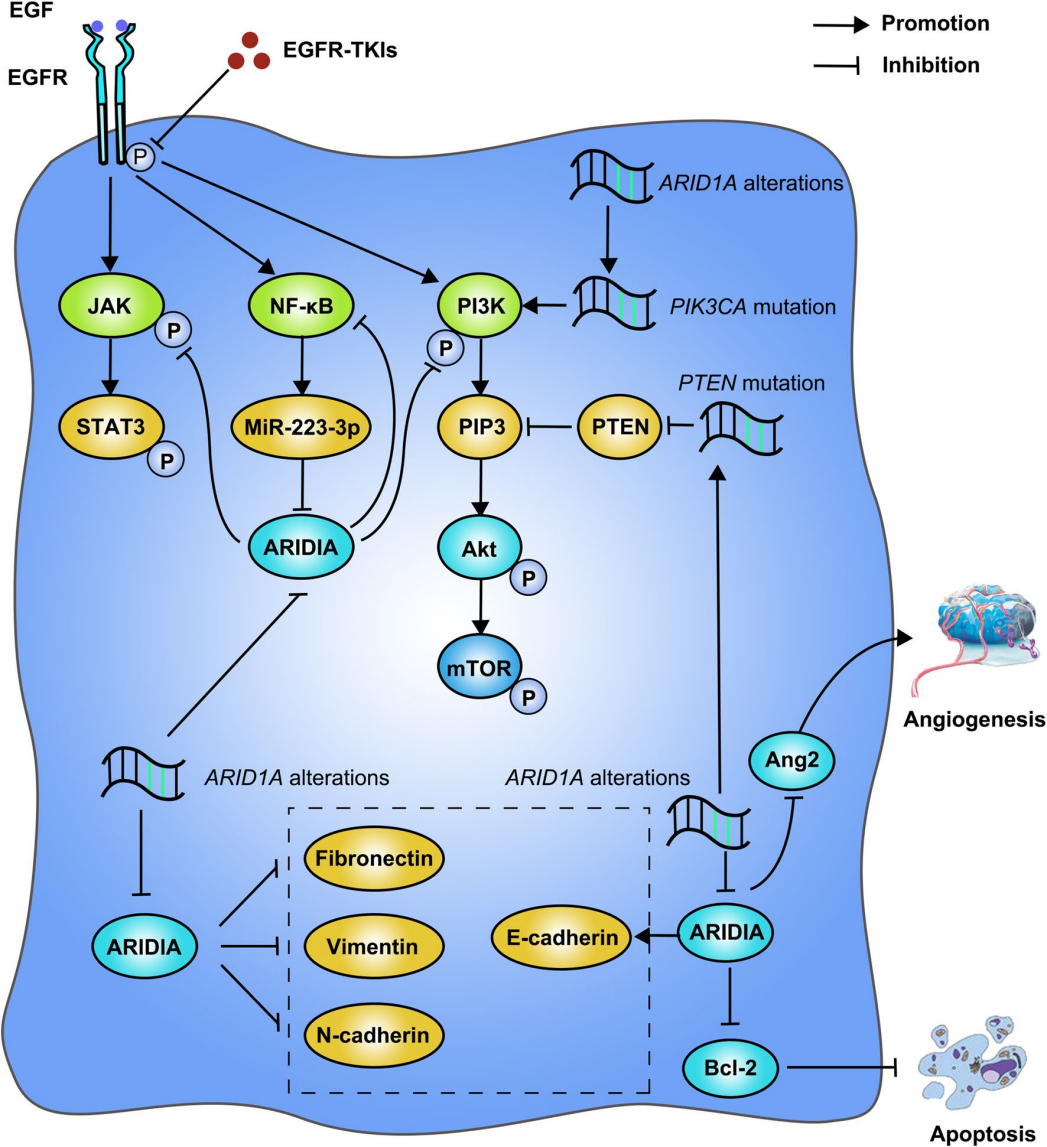
Mechanisms related to the resistance to EGFR-TKIs induced by *ARID1A* alterations or expression loss

In this review, we concluded a series of the published studies that focused on ARID1A in cancers and proposed the underlying mechanisms related to the resistance to EGFR-TKIs induced by *ARID1A* alterations or expression loss and the potential therapeutic strategies to overcome the resistance. It suggested that ARID1A might be the regulator in PI3K/Akt, JAK/STAT and NF-κB signaling pathways which are strongly associated with the resistance to EGFR-TKIs in NSCLC patients. Besides, *ARID1A* alterations or expression loss could contribute to the resistance to EGFR-TKIs via a variety of pathological process during tumor development, including epithelial to mesenchymal transition (EMT), angiogenesis of tumor and the inhibition of apoptosis. According to the potential mechanisms related to ARID1A, we summarized that the small molecular inhibitors targeting ARID1A or PI3K/Akt signaling pathway, the anti-angiogenic therapy and immunotherapy could be used as the supplementary treatment for EGFR-TKIs among NSCLC patients harboring the concomitant alterations of sensitive *EGFR* mutations and *ARID1A*. The mechanisms related to *ARID1A* alterations and expression loss in inducing the resistance to EGFR-TKIs are displayed in Fig. 1.

### Underlying mechanisms related to the resistance to EGFR-TKIs treatment in NSCLC induced by *ARID1A* alterations or expression loss

According to a latest study, Han et al. (2020) elucidated the potential role of *ARID1A* alterations in NSCLC patients harboring sensitive EGFR mutations. It suggested that *ARID1A* alterations are associated with the shorter progression free survival (PFS) of icotinib treatment for NSCLC patients (*P* = 0.001) and related to a higher level of phosphorylation of EGFR protein. Although previous research (Hung et al. 2020) discovered that *ARID1A* alterations or expression loss correlated with the reduction of the frequency of *EGFR* mutations, few studies had focused on the mechanisms of ARID1A alteration in inducing the insensitivity of EGFR-TKIs treatment. Therefore, we concluded the potential mechanisms as followed aim to clarify the role of ARID1A in EGFR-TKIs resistance and further explore the direction for research in this field.

### The activation of compensatory signaling pathways related to the resistance to EGFR-TKIs induced by *ARID1A* alterations or expression loss

#### 1. PI3K/Akt signaling pathway

The abnormal continuous activation of PI3K/Akt signaling pathway was believed to be one of most important signaling pathway resulting in the resistance to both first generation and third generation of EGFR-TKIs in NSCLC patients (Hrustanovic et al. 2013; Lin et al. 2014). The PI3K/Akt signaling pathway upregulates the phosphorylated level of downstream molecules of EGFR signaling pathway which endows the cancer cell the ability to escape from the inhibition of proliferation induced by EGFR-TKIs and could escape from the apoptosis which results in the development of the disease. In summary of the previous studies, it suggested that ARID1A is strongly believed to be the crucial trigger for the activation of PI3K/Akt signaling pathway. *ARID1A* alterations were found to be co-exist with a series of genes related to the PI3K/Akt signaling pathway especially including *PTEN* and *PIK3CA* (Samartzis et al. 2013; Takeda et al. 2016; Su et al. 2019). The aberrant of these genes was confirmed to reduce the inhibition of the pathway and result in the continuous activation. As for the expression loss of ARID1A, multiple studies had verified that the loss of ARID1A expression was also related to the alterations of genes belongs to PI3K/Akt signaling pathway (Bosse et al. 2013; Huang et al. 2014) and alternate the biological behaviors of tumor cells via this signaling pathway in variety of cancer types (Wiegand et al. 2014; Yang et al. 2019). Especially for the researches for lung cancer, Sun et al. (2021) and Zhang et al. (2014) further clarified the role of ARID1A expression loss respectively in the regulation of NSCLC behaviors via PI3K/Akt/mTOR signaling pathway in vitro and in vivo. It suggested ARID1A expression loss enhances the proliferation, metastasis and inhibits the apoptosis of NSCLC via this signaling pathway which contributed to the poor prognosis of NSCLC patients. To summarize the results above briefly, *ARID1A* alterations or expression loss could induce the resistance to EGFR-TKIs through the activation of PI3K/Akt signaling pathway.

#### 2. JAK/STAT signaling pathway

JAK/STAT signaling pathway serves as another important downstream pathway of activated EGFR signaling besides PI3K/Akt signaling pathway (Lin et al. 2014) and especially the activation of STAT3 pathway. According to the previous study performed by Alvarez et al. (2006), STAT3 pathway is a critical mediator of the oncogenic effects of somatic *EGFR* mutations and is necessary for the downstream phosphorylation in NSCLC while the inhibition of STAT3 signaling pathway significantly increased the apoptosis of tumor cells. The relationship between *ARID1A* alterations or expression loss with the activation of JAK/STAT signaling pathway, especially STAT3 signaling, had also been explored in the published researches. Peng et al. (2020) discovered the function of ARID1A expression loss as the regulator for the related genes of STAT3 signaling pathway and resulted in the impairment of apoptosis via the activated STAT3 signaling. In addition, Fang et al. (2015) proved that the ARID1A expression loss contributes to the tumorigenesis and development of HCC via activating the STAT3 signaling pathway and NF-κB signaling pathway. In this research, the authors constructed the HCC mouse models with *ARID1A* knockdown, and it demonstrated the rapid development of the disease compared with the control group. Therefore, we proposed that JAK/STAT signaling pathway plays an important role in the resistance to EGFR-TKIs induced by *ARID1A* alterations or expression loss. Nevertheless, it requires further clarification for this underlying mechanism.

#### 3. NF-κB signaling pathway

NF-κB signaling pathway is considered as the classical pivot signaling pathway related to tumorigenesis and development of malignancies and also confirmed to be associated with the resistance to EGFR-TKIs as described in previous studies (Hrustanovic et al. 2013; Cheong et al. 2018; Feng et al. 2018). As described above, ARID1A expression loss could activate the NF-κB signaling pathway and significantly change the biological behaviors of HCC (Fang et al. 2015). Besides, Kim et al. ( 2016) suggested that the inhibitors for NF-κB signaling pathway could reverse the resistance to chemotherapeutic drugs and suppress the proliferation conducted by the loss of ARID1A expression in ovarian clear cell carcinoma (OCCC). However, Yang et al. (2018) discovered the different pattern of ARID1A in participating in the NF-κB signaling pathway in their research. It suggested ARID1A serves as the downstream molecule of this pathway, NF-κB firstly stimulates the miR-223-3p expression which could directly bind to *ARID1A* and then influences the proliferation and migration of tumor cells through the function loss of ARID1A. To summarize the results above, ARID1A expression loss and NF-κB signaling pathway seem to be the feedback mechanism for the cancers and ARID1A expression loss could develop the resistance to EGFR-TKIs through this feedback mechanism.

### The promotion of EMT program induced by *ARID1A* alterations or expression loss

EMT program is believed as a crucial pathological process related to the development and metastasis of the malignancies and recent studies had confirmed its critical correlation with the resistance to EGFR-TKIs (Hrustanovic et al. 2013; Lin et al. 2014; Hou et al. 2019b). Besides the acquired metastatic tendency of stromal phenotypic tumor cells after EMT process, group of tumor cells could have the stem-cell like features through EMT process and escape from the inhibition of targeted drugs. The correlation between ARID1A and EMT had been found in previous studies. It suggested that *ARID1A* alterations are associated with the expression signature of EMT promoters related genes (Wilson et al. 2019). Furthers studies also confirmed the role of ARID1A expression loss in modulating the biomarkers for EMT process *through in vitro and in vivo experiments* (Wang et al. 2019,2020b; Somsuan et al. 2019; Tomihara et al. 2021). The expression loss of ARID1A upregulates the expression of fibronectin, vimentin and N-cadherin, while downregulates the expression of E-cadherin, which enables the transformation of the tumor cell phenotype to mesenchymal cell type characterized by the loss of cell polarity and the changes of cell morphology. Therefore, we strongly proposed that EMT program could participate into the resistance to EGFR-TKIs induced by *ARID1A* alterations or expression loss.

### Enhancement of tumor angiogenesis induced by *ARID1A* alterations or expression loss

The angiogenesis of tumor serves as another mechanism related to the resistance to EGFR-TKIs which nourishes the tumor cells and enables the cells to invade to the stroma and further metastasis (Alvarez et al. 2006). Recent studies had discovered the important function of ARID1A in regulating the process of angiogenesis. ARID1A expression loss was found tightly associated with the vessel density in solid tumor tissue (Hu et al. 2018) and more important, ARID1A expression loss causes the abnormal activation of angiopoietin-2 (Ang2) enhancer and promoter, which eventually leads to the ectopic expression of Ang2 (Hu et al. 2018; Yoodee et al. 2021), which is an essential molecule for angiogenesis process, and the resultant occurrence of the enhancement of angiogenesis. In addition, researchers confirmed that the blockage of Ang2 significantly reduced the density of vessels and the development of HCC with ARID1A deficiency (Hu et al. 2018). Evidences above suggest the underlying mechanism of EMT which is related to the resistance to EGFR-TKIs induced by *ARID1A* alterations or expression loss.

### Inhibition of the apoptosis induced by *ARID1A* alterations or expression loss.

It suggested that the expressions of B-cell lymphoma-2 (Bcl-2) family molecules play important roles in balancing the apoptosis and survive of tumor cells and the family was divided into two main group including apoptotic molecules, such as Bax, and anti-apoptotic molecules such as Bcl-2 and Bcl-XL. Specially, the overexpression of Bcl-2 resulting in the inhibition of the apoptosis which lead to the development of the disease and therapeutic resistance including EGFR-TKIs (Hou et al. 2019b). Through the review of the published studies, we discovered the role of *ARID1A* alterations or expression loss in the regulation of Bcl-2 expression and apoptosis of tumor cells which might participate into the resistance to EGFR-TKIs. It elucidated that loss of ARID1A expression could upregulate the expression of Bcl-2 and contribute to the inhibition of apoptosis of tumor cells (Zhang et al. 2018). Besides, researchers suggested that the tumor cells harboring *ARID1A* alterations showed the therapeutic sensitivity to Bcl-2 inhibitors which indicated the activation of apoptotic pathways induced by *ARID1A* alterations or expression loss.

### Strategies for overcoming the resistance to EGFR-TKIs induced by *ARID1A* alterations or expression loss

#### 1. Enhancer of zeste homolog 2 (EZH2) inhibitors

EZH2 is primarily an essential component of polycomb repressive complex 2 (PRC2) which serves a role in epigenetic gene suppression (Yamagishi and Uchimaru 2017). Latest reviews had concluded the role of EZH2 in the poor prognosis of a variety of cancers and the underlying potentiality of EZH2 inhibitors among cancer treatment (Yamagishi and Uchimaru 2017; Kim and Roberts 2016). In this review, we proposed that EZH2 inhibitors could be used in patients harboring *ARID1A* alterations or expression loss and serve as a potential option for the supplementary treatment of EGFR-TKIs. Firstly, EZH2 inhibitors is highly selective for the target of *ARID1A* alterations or expression loss. Bitler et al. (Bitler et al. 2015) confirmed that EZH2 inhibitors could significantly inhibit the proliferation of OCCC cells with altered *ARID1A* and either in cells with *ARID1A* knockdown. It suggested that ARID1A and EZH2 are a pair of important molecules in maintaining the balance of the proliferation and apoptosis of cells while ARID1A serves as the tumor suppressor. *ARID1A* alterations or expression loss leads to the advantage of EZH2 function and result in the excessive proliferation of tumor cells. Therefore, the purpose of EZH2 inhibition is to draw the balance of ARID1A and EZH2 back to the status before *ARID1A* alterations or expression loss which might reverse the resistance to EGFR-TKIs induced by *ARID1A* alterations or expression loss and have the synergistic interaction with EGFR-TKIs.

#### 2. MTOR inhibitors (rapamycin)

Previous studies established the role of mTOR inhibitors, especially rapamycin, in the treatment of NSCLC and its’ relationship with the administration of EGFR-TKIs. Kwon et al. (2019) proved that the the inhibition of the autophagy via targeting PI3K/Akt/mTOR signaling pathway could overcome the resistance to anti-EGFR treatment in NSCLC. Rolfo et al. (2014) also elucidated that rapamycin could serve as an option for NSCLC patients harboring sensitive *EGFR* mutations that do not response to EGFR-TKIs. As described above, PI3K/Akt/mTOR signaling pathway serve as the main mechanism related to the resistance to EGFR-TKIs induced by *ARID1A* alterations and expression loss in NSCLC patients. Therefore, rapamycin might benefit the NSCLC patients harboring the concomitant alterations of *EGFR* and *ARID1A*.

#### 3. Anti-angiogenic therapy

As far as we concerned, *ARID1A* alterations or expression loss could upregulate the expression of Ang2 and initiate the process of angiogenesis (Hu et al. 2018; Yoodee et al. 2021). In addition, Hu et al. (2018) revealed that the blockage of Ang2 could reverse the change of tumor behaviors induced by *ARID1A* alterations or expression loss which uncovers the potentiality of anti-angiogenic therapy in overcoming the resistance to EGFR-TKIs, such as the treatment of EGFR-TKIs combined with bevacizumab.

#### 4. Immune checkpoint inhibitors (ICIs)

ICIs had been wildly used in the treatment of cancers recently. According to latest researches, ARID1A was found to be related to the sensitivity of ICIs treatment. Li et al. (2020) disclosed that the intact ARID1A contributes to the chromatin accessibility and expression to IFN-responsive genes which eventually influence the infiltration of lymphocytes. It suggested that ARID1A has the function of modulating the immune phenotype of cancers. Other studies also pointed that cancer patients harboring *ARID1A* alterations could benefit from ICIs treatment (Goswami et al. 2020; Takahashi et al. 2021). Although NSCLC patients harboring driver mutations such as *EGFR* was considered as the group that might not benefit from ICIs, the concomitant alterations of *EGFR* and *ARID1A* might reverse the consequences of the treatment.

#### 5. Other underlying targets

Latest studies discovered several novel targets for the treatment of *ARID1A*-mutated cancers. It suggested that the inhibition of GLS1 (Wu et al. 2021) or CCNE1 (Kawahara et al. 2021) could significantly suppress the proliferation of *ARID1A*-mutated cancer cells in vitro and in vivo, respectively, but not in the wild type cells. Another study also confirmed that the inhibition of ATM/Chk2 DNA damage checkpoint axis would exhibit anti-cancer efficacy only in *ARID1A*-mutated cancer cells (Wang et al. 2020c). Targets above would provided us with more options for the treatment of *ARID1A*-mutated cancer but requires further studies.

## Conclusion

ARID1A is the regulator a series of signaling pathways, including PI3K/Akt, JAK/STAT and NF-κB signaling pathway and related to the resistance to EGFR-TKIs in NSCLC patients. Besides, *ARID1A* alterations or expression loss could lead to the resistance to EGFR-TKIs via enhancing the EMT, angiogenesis and the inhibition of apoptosis in NSCLC. In order to overcome the resistance to EGFR-TKIs related to ARID1A, EZH2 inhibitor, rapamycin and the anti-angiogenic therapy could be used for the supplementary treatment for NSCLC patients that do not response to EGFR-TKIs.

## Data Availability

Not applicable.

## Abbreviations

SCLC: Small cell lung cancer
NSCLC: Non-small-cell lung cancer
EC: Endometrial carcinoma
EGFR: Epidermal growth factor receptor
ALK: Anaplastic lymphoma kinase
IGF1R: Insulin-like growth factor 1 receptor
HGF: Hepatocyte growth factor
SWI/SNF: Switch/sucrose nonfermenting
ARID1A: AT-rich interactive domain 1A
DSBs: DNA double-strand breaks
EMT: Epithelial to mesenchymal transition
PFS: Progression free survival
HCC: Hepatocellular carcinoma
OCCC: Ovarian clear cell carcinoma
Ang2: Angiopoietin-2
Bcl-2: B-cell lymphoma-2
EZH2: Enhancer of zeste homolog 2
ICIs: Immune checkpoint inhibitors
